# The essential function of CARD9 in diet‐induced inflammation and metabolic disorders in mice

**DOI:** 10.1111/jcmm.13494

**Published:** 2018-03-25

**Authors:** Xuejiao Zeng, Xihao Du, Jia Zhang, Shuo Jiang, Jie Liu, Yuquan Xie, Wei Shan, Guanglong He, Qinghua Sun, Jinzhuo Zhao

**Affiliations:** ^1^ Department of Environmental Health School of Public Health Fudan University Shanghai China; ^2^ The Key Laboratory of Public Health Safety Ministry of Education Fudan University Shanghai China; ^3^ Department of Cardiology School of Medicine Xinhua Hospital Shanghai Jiao Tong University Shanghai China; ^4^ Department of Epidemiology School of Public Health Fudan University Shanghai China; ^5^ Ministry of Education Fudan University Shanghai China; ^6^ College of Health Sciences University of Wyoming School of Pharmacy Laramie WY USA; ^7^ Division of Environmental Health Sciences College of Public Health The Ohio State University Columbus OH USA; ^8^ Shanghai Key Laboratory of Meteorology and Health Shanghai China

**Keywords:** CARD9, energy metabolism obesity, insulin resistance, inflammation

## Abstract

Inflammation and metabolic disorder are common pathophysiological conditions, which play a vital role in the development of obesity and type 2 diabetes. The purpose of this study was to explore the effects of caspase recruitment domain (CARD) 9 in the high fat diet (HFD)‐treated mice and attempt to find a molecular therapeutic target for obesity development and treatment. Sixteen male CARD9^−/−^ and corresponding male WT mice were fed with normal diet or high fat diet, respectively, for 12 weeks. Glucose tolerance, insulin resistance, oxygen consumption and heat production of the mice were detected. The CARD9/MAPK pathway‐related gene and protein were determined in insulin‐responsive organs using Western blotting and quantitative PCR. The results showed that HFD‐induced insulin resistance and impairment of glucose tolerance were more severe in WT mice than that in the CARD9^−/−^ mice. CARD9 absence significantly modified O_2_ consumption, CO_2_ production and heat production. CARD9^−/−^ mice displayed the lower expression of p38 MAPK, JNK and ERK when compared to the WT mice in both HFD‐ and ND‐treated groups. HFD induced the increase of p38 MAPK, JNK and ERK in WT mice but not in the CARD9^−/−^ mice. The results indicated that CARD9 absence could be a vital protective factor in diet‐induced obesity via the CARD9/MAPK pathway, which may provide new insights into the development of gene knockout to improving diet‐induced obesity and metabolism disorder.

## Introduction

Caspase recruitment domain (CARD) 9 is an important adapter protein that was originally recognized through a database search for CARD‐containing proteins, which is widely expressed in plentiful tissues including liver, spleen, bone marrow, brain, lung and peripheral blood 1, particularly in the macrophages, neutrophil granulocytes and dendritic cells 2. CARD9 is closely associated with immune responses, immune cell activation 3, 4 and inflammatory response 5. CARD9 utilizes B‐Cell lymphoma 10 (BCL10) and MALT1 to mediate Dectin‐1 signalling for the activation of nuclear factor‐k‐gene binding (NF‐κB), p38 mitogen‐activated protein kinase (p38 MAPK), and c‐Jun N‐terminal kinase (JNK) 2, 3. Previous studies have demonstrated that overexpression of CARD9 strongly activates the kinases p38 and JNK, both of which are pivotal in immune responses and the production of pro‐inflammatory cytokines 3, 4, 6. Moreover, it has reported that CARD9 is associated with a member of Ras family of small guanosine triphosphatases (H‐Ras) and phosphorylated Ras‐guanine‐nucleotide‐releasing factor 1(Ras‐GRF1), leading to the activation of extracellular signal‐regulated kinase (ERK) pathway 7.

Stimulation of dendritic cells in CARD9^−/−^ mice with the cell wall component zymosan or whole C. albicans cells results in a decrease in the release of inflammatory cytokines 8 including interleukin (IL)‐2, IL‐6, IL‐10 and tumour necrosis factor‐α (TNF‐α) 3, 5. Recently, studies have focused on defining the precise molecular mechanism of CARD9 signalling and exploring its role in the development of non‐infectious disease including cardiovascular diseases 9, 10. Obesity and type 2 diabetes (T2D) have become the leading health problem in the world. Low‐grade systemic inflammation and immune disorders are linked to the development of these complications 11, 12. It is reported that obese patients skewed towards pro‐inflammatory subsets 13. Obesity originated from high fat diet (HFD) has been linked to a pro‐inflammatory state, which in turn leads to the development of metabolic syndrome. Because of the vital role of CARD9 in inflammatory response, several studies have reported that CARD9/MAPK signalling pathway plays an important role in diet‐induced myocardial dysfunction 14, 15. However, whether or not there is a link between CARD9 signalling and obesity‐associated metabolic disorder is unknown. We attempt to explore the function of CARD9/MAPK pathway in diet‐induced insulin resistance, glucose tolerance impairment, insulin‐responsive organs and brown adipose inflammation. In this study, CARD9^−/−^ and C57BL/6 mice were fed with HFD to establish obese animal model to evaluate the impact of CARD9 on diet‐induced inflammation and metabolic disorders, and to explore the potential mechanism of CARD9 in mediating energy metabolism.

## Materials and methods

### Mice

The CARD9^−/−^ mice were kindly provided by Professor Xin Lin (Department of Molecular and Cellular Oncology, University of Texas, Houston, TX, USA). The background of the mice is C57BL/6. Sixteen six‐week‐old male CARD9^−/−^ mice were randomly divided into two groups: CARD9^−/−^‐normal diet (ND) group and CARD9^−/−^‐HFD group, which were fed with HD or HFD, respectively. HFD (Shanghai Laboratory Animal Centre, Shanghai, China) is a mixture of 18.9% protein, 44.6% carbohydrate and 36.5% kcal/g fat and was known to induce obesity and diabetes. Similarly, sixteen six‐week‐old male wide‐type (WT) C57BL/6 mice were purchased from Laboratory Animal Centre of Fudan University. The mice were randomly divided into two groups (WT‐ND group and WT‐HFD group) and given ND or HFD, respectively. All the mice were housed in standard cages with a constant temperature range of 22 ± 2°C, 60% relative humidity and an artificial 12‐hr light/dark cycle. The study conforms to the Guide for the Care and Use of Laboratory Animals published by the US National Institutes of Health (NIH Publication No. 85‐23, revised 1996), and the protocols were approved by the Institutional Animal Care and Use Committee at Fudan University.

### Glucose tolerance and insulin sensitivity determination

At week 8 and 12, glucose tolerance test (GTT) and insulin tolerance test (ITT) were performed to observe the energy metabolism. The mice were fasted 6 hr and intraperitoneally injected dextrose (2 mg/g b.w.) for GTT. Blood samples were collected from caudalis vena, and blood glucose was measured with a Contour Blood Glucose Meter (Bayer, Ezreal, NJ, USA) at baseline, 30‐, 60‐, 90‐ and 120 min after the dextrose injection. Insulin levels were determined using ultra‐sensitive mouse insulin ELISA kit (R&D Systems, Minnesota, MN, USA) according to the manufacturer's instruction. In addition, insulin sensitivity was measured by ITT. The method is as follows: after 4.5 hr fasting, insulin (0.5 U/kg b.w.) was administered intraperitoneally, and blood glucose measurement was conducted in the same way as GTT after insulin injection. The homeostasis model assessment‐estimated insulin resistance (HOMA‐IR) was calculated based on 1 mg of insulin as equivalent to 24 IU, using the formula HOMA‐IR = [fasting insulin concentration (mU/l) × fasting glucose concentration mmol/l)]/22.5 16.

### Metabolic cage

Oxygen consumption, carbon dioxide production and heat production were measured by metabolic cage. The mice were isolated in a semi‐sealed cage, and the inner air was aspirated at a constant volume/min. VO_2_, VCO_2_, respiration exchange ratio(RER), and heat production were measured simultaneously using a computer‐controlled analyzers (TSE‐System, Bad Homburg, Germany). Each mouse was measured individually in a resting state at 22°C in the presence of food and water 17. Measurements were undertaken for a period of 24 hr (from 9:19 a.m. to 9:19 a.m. the next day), including a 12 hr light cycle and a 12 hr dark cycle. Data were normalized to body weight.

### Tissue and blood collection

After 12 weeks of HFD and ND treatment, the CARD9^−/−^ and WT mice were killedand blood samples were collected, which were then subjected to the centrifugation (4°C) at 1006 × g for 15 min. Supernatant was collected and stored at −80°C immediately. The spleen, liver, white adipose tissue (WAT: epididymal adipose, inguinal adipose, perinephric adipose) and brown adipose tissue (BAT) were harvested, and the tissue weights were measured. Liver, WAT and BAT were immediately frozen at −80°C, and parts of the tissues were fixed in 4% paraformaldehyde for pathological examination.

### Histological analyses

Liver, WAT and BAT were fixed in 4% paraformaldehyde, embedded in paraffin, cut into 4 μm‐thick tissue sections, and stained with haematoxylin & eosin (H&E). Microscopic examination was implemented, and photographs were taken under light microscope (Nikon Eclipse TE2000‐U; Nikon, Tokyo, Japan). From each section, ten random areas were examined at a magnification of ×400 for liver and BAT, and ×200 for WAT. The slides were graded independently in a masked fashion by two observers.

### Inflammatory cytokine determination in serum

The expressions of IL‐6 and TNF‐α in serum were determined using ELISA kits. The tests were performed strictly according to the manufacturer's instructions.

### Quantitative real‐time PCR analysis

Quantitative real‐time PCR was used to determine the mRNA expression. Total RNA from liver and WAT were isolated with trizol (Thermo Fisher Scientific, Waltham, MA, USA) according to the manufacturer's protocol. One ug of total RNA was applied for cDNA synthesis, and cDNA was reversely transcribed using Revertid^™^ First Strand cDNA Synthesis Kit (Takara Bio Company, Sigma, Japan). Primers were designed according to the primer design principles. The mRNA expressions for glyceraldehyde phosphate dehydrogenase (GAPDH), IL‐6, TNF‐α, JNK, ERK1/2, MAPK‐p38, and NF‐κB were determined using inventoried primer and probe assays on an ABI 7500 Real‐Time PCR System (Applied Biosystems, Waltham, MA, USA), and using SuperReal PreMix Plus (SYBR Green) fluorescence (Tiangen biotech, Beijing, China). The universal two‐step RT‐PCR cycling conditions were performed in a total volume of 25 μl. The 2^−▵▵CT^ method 17 was used to normalize transcription to GAPDH mRNA, and the relative mRNA expression was calculated.

### Western blot analysis

Protein levels were determined by Western blot. Liver and BAT were homogenized with M‐PER mammalian protein extraction reagent (Thermo Scientific, Waltham, MA, USA) on ice. Equal quantities of protein, respective of tissue quantities, were separated by 10% sodium dodecyl sulphate‐polyacrylamide gel electrophoresis (SDS‐PAGE). Following transfer to immobilon‐P polyvinylidene difluoride (PVDF) membrane and blocking with 5% nonfat milk or 5% BSA, the blot was incubated with different primary antibodies (Cell Signalling Technology, Danvers, MA, USA): uncoupling protein 1 (UCP1)(1:1000), p‐JNK (1:1000), JNK (1:1000), p38 (1:1000), p‐p38 (1:1000), NF‐κB (USA, 1:1000), p‐NF‐κB (Cell 1:1000), ERK1/2(1:1000), p‐ERK1/2(1:2000) at 4°C overnight, and then incubated with secondary HRP‐conjugated antibody (1:5000; Kangchen Biotech, Shanghai, China) for 2 hr at room temperature. After incubation with the secondary antibody, the membranes were detected with enhanced chemiluminescence followed by exposure to X‐ray film. The protein bands on the X‐ray film were scanned, and band density was calculated by software Quantity one 4.6.2 (Bio‐Rad Software Inc., Hercules, CA, USA). β‐actin and GAPDH were used as loading control reference, respectively.

### Statistical analysis

Data are expressed as means ± standard deviation (S.D.). SPSS 19.0 IBM statistical software was used for the analysis. One‐way analysis of variance (anova) was used to compare the difference among the four groups. The *P* value <0.05 was considered statistically significant.

## Results

### Body weight, food intake and organ weight measurements

**Figure 1:**
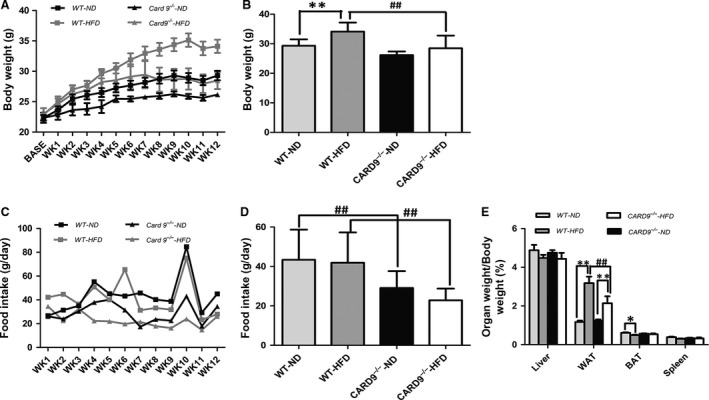
Changes in body weight (b.w.), food intake and the ratio of organ/b.w in the CARD9^−/−^ and WT mice after ND and HFD feeding. Weekly changes of b.w. (**A**), b.w. at the end of 12 weeks feeding (**B**), Weekly food intake (**C**),Average food intake during the entire experiment (**D**), The ratios of organ/b.w. in liver, WAT, BAT and spleen (**E**). *P < 0.05, **P < 0.01, HFD *vs*. ND; ^#^ *P* < 0.05, ^##^ *P* < 0.01, CARD9^−/−^ *vs*. WT.

Figure 1A illustrated that baseline body weight was no significant statistical difference among the four groups, and the body weight of the mice was gradually increased with time. As shown in Figure 1B, HFD induced the increase of body weight both in WT and CARD9^−/−^ mice, but there is no statistical difference between ND‐treated and HFD‐treated CARD9^−/−^ mice. Similarly, there was no difference in food intake between ND and HFD groups in both CARD9^−/−^ and WT mice. The food intake of CARD9^−/−^ mice was significantly lower than that in WT mice when fed with ND or HFD (*P* < 0.05; Fig. 1C and D). The ratios of liver, spleen, WAT and BAT weights to body weight were also analysed. As displayed in Figure 1E, there was no statistical difference of the liver weight/b.w. and spleen weight/b.w. among the four groups. The ratio of BAT/b.w. was higher in the ND‐treated WT mice than that in the HFD‐treated WT mice. The ratio of WAT/b.w. in the WT mice fed with HFD was higher than that in the mice fed with ND (*P* < 0.05). However, there was no difference of WAT/b.w between the HFD‐ and ND‐CARD9^−/−^ mice.

### Effect of CARD9 on glucose homeostasis and insulin sensitivity

**Figure 2:**
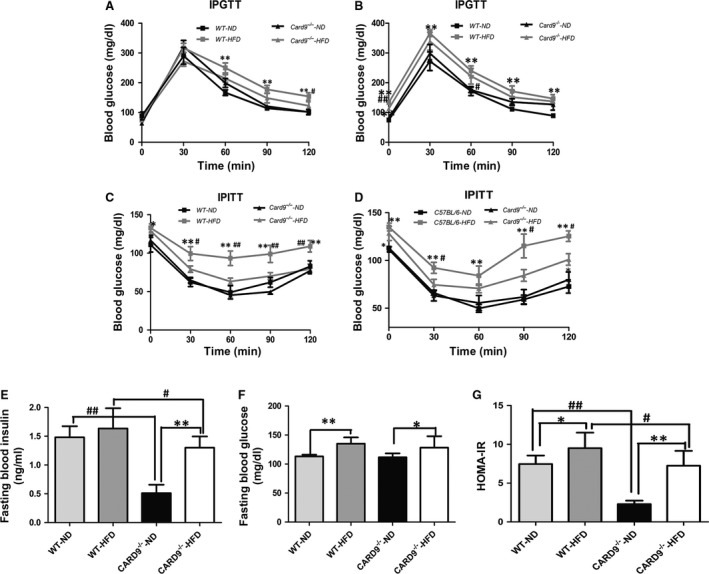
Glucose homeostasis and insulin sensitivity in the CARD9^−/−^ and WT mice at week 8 and 12. IPGTT at week 8 (**A**) and week 12 (**B**), IPITT at week 8 (**C**) and week 12 (**D**), Fasting blood glucose level (**E**), Fasting blood insulin level (**F**), HOMA‐IR index (**G**). *P < 0.05, **P < 0.01, HFD *vs*. ND; ^#^ *P* < 0.05, ^##^ *P* < 0.01, CARD9^−/−^ *vs*. WT.

To determine if there was an effect of CARD9 on diet‐induced glucose homeostasis and insulin sensitivity, IPGTT and ITT were measured at week 8 and 12, respectively (Fig. 2). As shown in Figure 2A, HFD feeding induced the impairment of the glucose tolerance in C57BL/C mice but not in CARD9^−/−^ mice when compared with corresponding ND‐fed mice. Meanwhile, WT‐HFD displayed more severe impairment of the glucose tolerance (Fig. 2A). Figure 2B showed the glucose tolerance at week 12. The results indicated that the baseline glucose has a significant increase in HFD‐fed groups, which suggested that the effect was more significant at week 12 than that at week 8 (Fig. 2B). IPITT results showed that HFD feeding impaired insulin utilization especially in C57BL/C mice. HFD‐fed mice demonstrated rapidly elevations in glucose levels at all time points. However, there is no significant difference between HFD‐fed CARD9^−/−^ mice and ND‐fed CARD9^−/−^ mice at week 8 (Fig. 2C). Intriguing, the insulin sensitivity became worse at week 12 in HFD‐fed CARD9^−/−^ mice when compared with ND‐fed CARD9^−/−^ mice (Fig. 2D). In addition, fasting blood insulin and fasting blood glucose levels in serum were increased in HFD‐fed mice compared to ND‐fed mice (*P* < 0.05; Fig. 2E and F). The HFD‐fed CARD9^−/−^ mice displayed significant increase in fasting blood insulin level when compared to the ND‐fed CARD9^−/−^ mice, but not in WT mice (Fig. 2E). HOMA‐IR index, an indicator of insulin sensitivity 18, was significantly higher in the HFD‐fed mice than that in the ND‐fed mice (*P* < 0.05; Fig. 2G). Furthermore, compared to the WT mice, CARD9^−/−^ mice had lower HOMA‐IR.

### Oxygen consumption and heat production measurement

**Figure 3:**
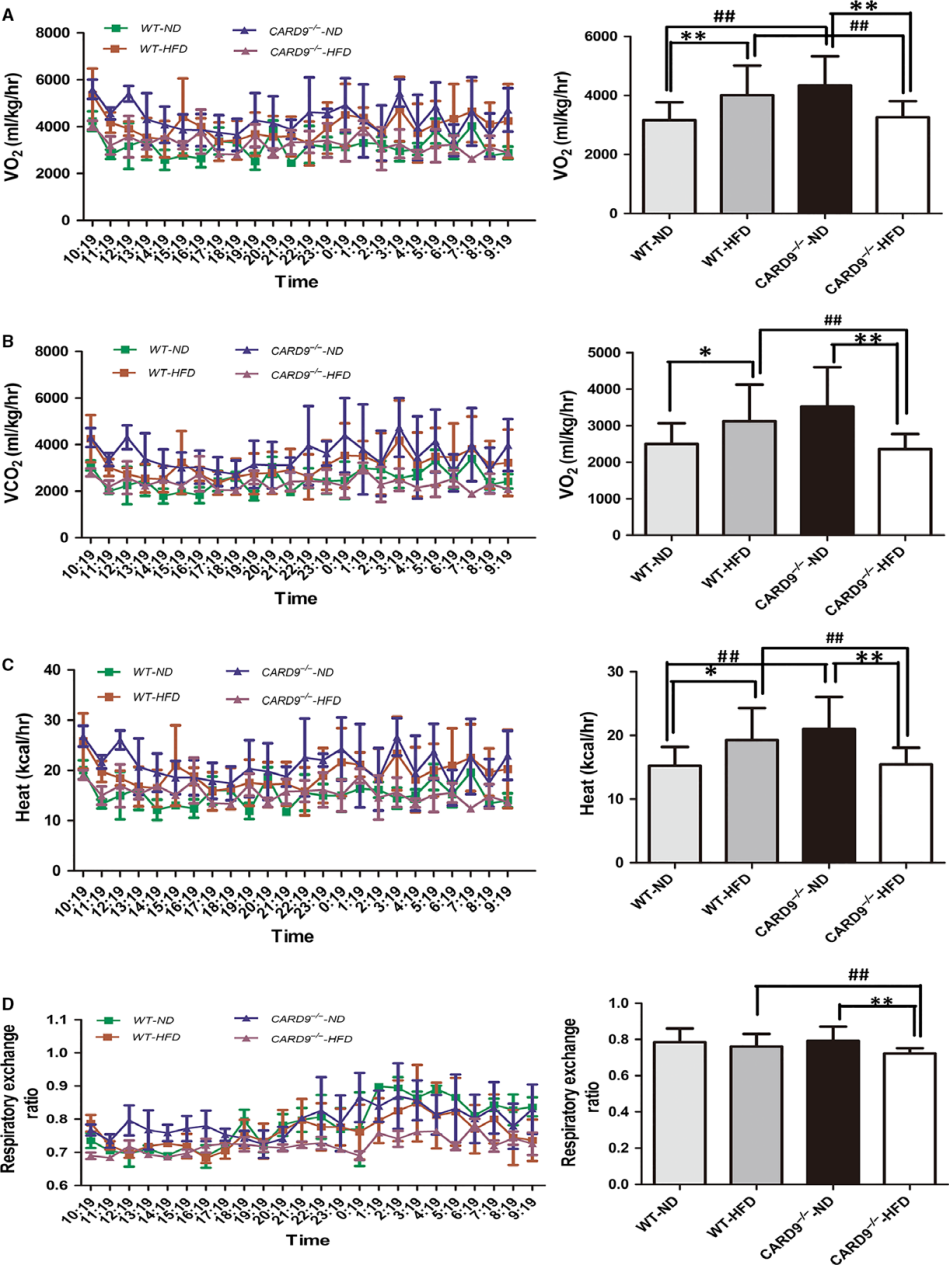
Energy homeostasis in the CARD9^−/−^ and WT mice after ND and HFD feeding. O_2_ consumption (**A**), CO_2_ consumption (**B**), Heat production (**C**), Respiratory exchange ratio (**D**). Left panel: the trends of above four indicators with time, Right panel: the bar graph of above four indicators in the four groups, respectively. *P < 0.05, **P < 0.01, HFD *vs*. ND; ^#^ *P* < 0.05, ^##^ *P* < 0.01, CARD9^−/−^ *vs*. WT.

The energy homeostasis was determined in WT and CARD9^−/−^ mice (Fig. 3). As shown in Figure 3, VO_2_ (Fig. 3A), VCO_2_ (Fig. 3B), heat production (Fig. 3C) and RER (Fig. 3D) are higher in WT‐HFD group than that in WT‐ND group, but it was contrary in the CARD9^−/−^ mice. That means that the HFD‐fed CARD9^−/−^ mice showed lower VO_2_, VCO_2_, heat production and RER when compared to the ND‐fed CARD9^−/−^ mice. Interestingly, CARD9^−/−^‐ND mice showed significantly higher VO_2_, VCO_2_, heat production and RER when compared to WT‐ND mice (*P* < 0.05). However, CARD9^−/−^‐HFD group showed significantly lower VO_2_, VCO_2_, heat production and RER compared to WT‐HFD mice (*P* < 0.05). As shown in Figure 3D, compared to the ND‐treated group, HFD caused a decrease of RER in CARD9^−/−^ mice. Moreover, there was a lower RER in CARD9^−/−^‐HFD group compared to the WT‐HFD group.

### Peripheral inflammation in serum

**Figure 4:**
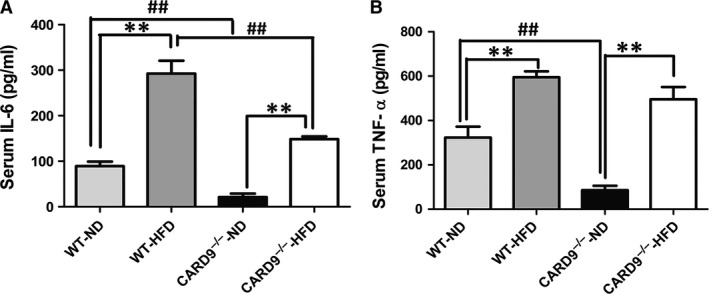
Systemic inflammation in the CARD9^−/−^ and WT mice after ND and HFD feeding. IL‐6 (**A**), TNF‐α (**B**). *P < 0.05, **P < 0.01, HFD *vs*. ND; ^#^ *P* < 0.05, ^##^ *P* < 0.01, CARD9^−/−^ *vs*. WT.

Serous IL‐6 and TNF‐α were detected in mice. Figure 4A and B illustrated that the levels of IL‐6 in HFD‐fed mice were significantly higher compared to that of ND‐fed mice (*P* < 0.05). In addition, compared to the WT mice, CARD9 deficiency significantly decreased the levels of IL‐6 in both HFD and ND group. Similarly, HFD induced the increase of TNF‐α in both CARD9^−/−^ and WT mice. There is a significant difference of TNF‐α between CARD9^−/−^‐ND and WT‐ND mice (*P* < 0.05), but there is no statistical difference between CARD9^−/−^‐HFD and WT‐HFD mice although there is a decrease of TNF‐α in CARD9^−/−^‐HFD mice compare to the WT‐HFD mice.

### Histological analyses

**Figure 5:**
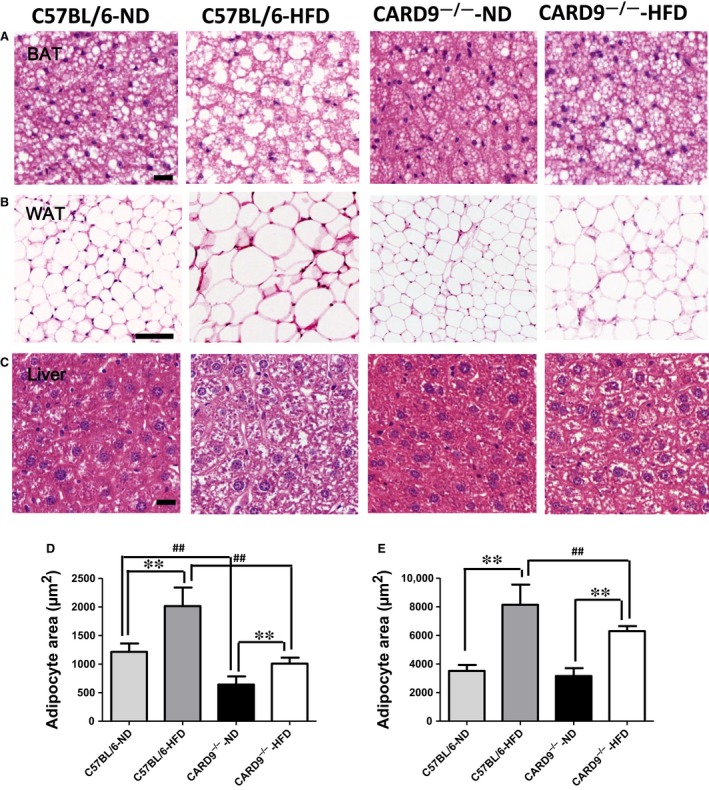
Representative histological images of liver, white adipose fat (WAT) and brown adipose fat (BAT) stained with haematoxylin and eosin (H&E). BAT (**A**), original magnification, ×400, scale bar = 100 μm; WAT (**B**), original magnification, ×200, scale bar = 100 μm; Liver (**C**), original magnification, ×400, scale bar = 100 μm. Adipocyte area was calculated from 100 adipocytes of BAT (**D**) and WAT (**E**) in each mouse.*P < 0.05, **P < 0.01, HFD *vs*. ND; ^#^ *P* < 0.05, ^##^ *P* < 0.01, CARD9^−/−^ *vs*. WT.

Histological analyses were used to observe the morphology of liver, WAT and BAT in the WT and CARD9^−/−^ mice (Fig. 5). As shown in Figure 5, the mean diameter of the adipocytes of WAT and BAT was larger in the HFD‐fed mice when compared to the ND‐fed mice. Furthermore, the results (Fig. 5D and E) showed that the adipocyte size of WAT and BAT was smaller in CARD9^−/−^ mice when compared to the WT mice (*P* < 0.05). Compared to the ND‐fed mice, there was significant inflammatory cell infiltration, vacuolation and extensive hepatocyte swelling in the mice fed with HFD, whereas CARD9 knockout may ameliorate the cell swell.

### The mRNA expression of CARD9/MAPK pathway in liver and WAT

**Figure 6:**
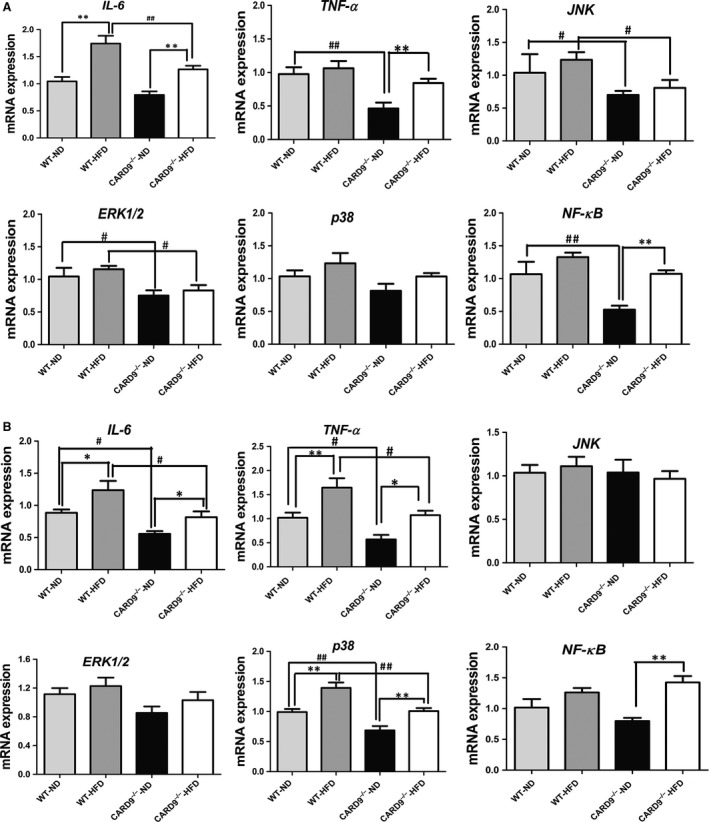
mRNA expressions of IL‐6, TNF‐α, p38, ERK1/2, JNK and NF‐κB in the CARD9^−/−^ and WT mice after ND and HFD feeding. The expression in liver (**A**), WAT (**B**). *P < 0.05, **P < 0.01, HFD *vs*. ND; ^#^ *P* < 0.05, ^##^ *P* < 0.01, CARD9^−/−^ *vs*. WT.

To assess the alterations of liver‐ and adipose‐related gene expression in HFD‐induced inflammation between WT and CARD9^−/−^ mice, the mRNA expression of IL‐6, TNF‐α, JNK, ERK1/2, p38 and NF‐κB in liver (Fig. 6A) and WAT (Fig. 6B) were measured by quantitative PCR. As shown in Figure 6A, compared to the WT mice, the CARD9^−/−^ mice displayed lower expression of IL‐6, TNF‐α, JNK, ERK1/2 and NF‐κB in both ND‐fed group and HFD‐fed group (*P* < 0.05). HFD feeding induced the increase of IL‐6, TNF‐α, JNK, ERK1/2 and NF‐κB although some have no statistical significance, whereas CARD9 deficiency reduced the elevation of these genes expression especially in IL‐6, TNF‐α, JNK, ERK and NF‐κB. In addition, in CARD9^−/−^ mice, HFD feeding induced the increase of IL‐6, TNF‐α and NF‐κB when compared with the corresponding ND group (*P* < 0.05); however, HFD treatment did not induce the statistical increase in JNK, ERK1/2 and p38 expression in CARD9^−/−^ mice.

Similarly, the mRNA expressions of the genes were detected in WAT (Fig. 6B). The results indicated that the expressions of IL‐6, TNF‐α, p38 and NF‐κB were higher in the HFD‐fed group when compared to the ND‐fed group in both CARD9‐/‐ mice and WT mice (*P* < 0.05). Moreover, CARD9^−/−^ mice had a lower expression of IL‐6, TNF‐α and p38 when compared to the WT mice both in HFD‐ and ND‐fed groups (*P* < 0.05). Figure 6B also showed that the mRNA expression of NF‐κB in CARD9^−/−^‐HFD group is remarkably higher than that in CARD9^−/−^‐ND group. In contrast, the mRNA expression of JNK and ERK did not show statistical difference among the four groups in WAT.

### The protein expression of CARD9/MAPK pathway

**Figure 7:**
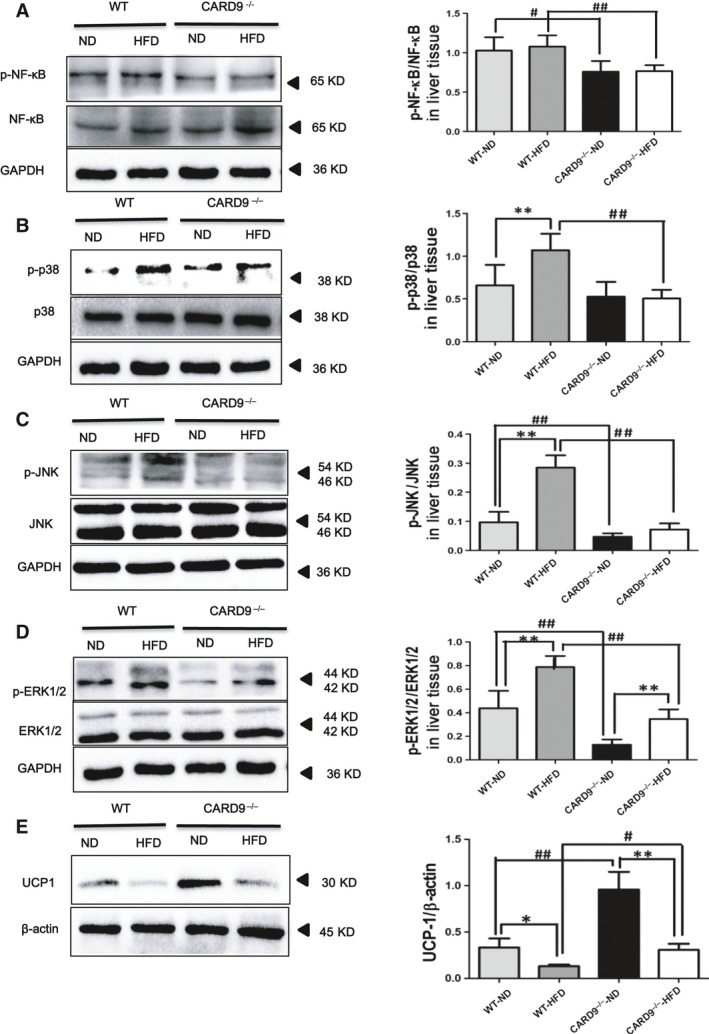
Protein expression of inflammation and MAPK signalling pathways in the liver of the CARD9^−/−^ and WT mice after ND and HFD feeding, along with the UCP‐1 expression in BAT. Western blotting for phosphorylated NF‐κB (P‐NF‐κB)/total NF‐κB (**A**). Western blotting of signalling molecules involved in MAPK pathway [P‐p38/p38, (**B**); P‐JNK/JNK, (**C**); P‐ERK/ERK, (**D**)]. Western blotting of UCP‐1 (**E**). *P < 0.05, **P < 0.01, HFD *vs*. ND; ^#^ *P* < 0.05, ^##^ *P* < 0.01, CARD9^−/−^ *vs*. WT.

Figure 7 lists the protein expression of p38, JNK, ERK1/2 and NF‐κB in liver. Meanwhile, the protein expression of UCP‐1 was determined in BAT. The CARD9^−/−^ mice exhibited a significantly lower expression of NF‐κB (Fig. 7A), p38 (Fig. 7B), JNK (Fig. 7C) and ERK1/2 (Fig. 7D) when compared to the WT mice (*P* < 0.05). HFD induced the significant elevations of p38 and JNK in WT mice but not in CARD9^−/−^ mice, which suggested that HFD‐induced p38 and JNK activation was diminished in the absence of CARD9. However, HFD induced the increase of ERK1/2 in both WT mice and CARD9^−/−^ mice. There are no significant changes in NF‐κB between HFD‐fed and ND‐fed groups.

UCP1 is largely responsible for the uncoupling of respiration from ATP synthesis, which is specifically expressed in BAT. In this study, CARD9^−/−^ mice displayed a higher expression of UCP‐1 in BAT than that in the WT mice (*P* < 0.05). HFD caused the reduction in UCP‐1 expression both in WT mice and in CARD9^−/−^ mice, but CARD9 deficiency could inhibit HFD‐induced reduction in UCP‐1 (Fig. 7E).

## Discussion

In this study, we demonstrated that the CARD9‐associated NF‐κB and MAPKs signalling pathway played an important role in diet‐induced inflammatory response, metabolic disorders, insulin resistance and glucose tolerance impairment. CARD9 knockout may ameliorate the metabolic disorders and inflammatory response in insulin susceptible organs in mice after 8–12 weeks of HFD feeding.

Obesity is a major clinical risk factor for T2D and cardiovascular diseases. Previous studies have explored the association between inflammatory response 19, immune injury 20 and T2D. Energy disorder, insulin resistance and inflammation are the major events of diet‐induced injury, which are the major risk factors in the development of T2D. Therefore, it is critical to find effective therapies to prevent diet‐induced health outcome 21. Although a general consensus concerning the role of CARD9 expression in infectious diseases has been reached 3, 22, 23, its role in diet‐induced inflammation and metabolic disorders is still less obvious.

In this study, body weight, WAT weight/b.w and food intake are lower in HFD‐CARD9^−/−^ mice than that in HFD‐WT mice, suggesting that CARD9 knockout reduced the excessive fat accumulation. Along with the higher VCO_2_, VO_2_, RER, and heat production in ND‐CARD9^−/−^ mice when compared to ND‐WT mice, suggesting that CARD9 deficiency increased the energy metabolism and could diminish the fat accumulation under normal circumstances. After fed with HFD, WT mice displayed a significant increase of VCO_2_, VO_2_, RER and heat when compared with the ND group, but CARD9^−/−^ mice displayed a contrary result. This very strange result might be due to the more severe metabolic disorders in WT mice than that in CARD9^−/−^ mice after fed with HFD, which is consistent with common clinical outcome that patients with severe metabolic disorders displayed a high release of heat, but there is a low release of heat in the early slight metabolic disorders. In contrast, in this study, HFD‐WT mice and HFD‐CARD9^−/−^ mice might represent the severe and slight metabolic disorders, respectively. Arimura *et al*. 24 reported that high protein diet‐fed *db* mice showed significantly higher water intake, urinary volume and glucose levels than the low protein diet‐fed *db* mice, which supported our results. In addition, clinical study found that the late stages of metabolic disease (such as diabetic kidney disease and nonalcoholic fatty liver disease) appeared the production of more heat, increase of metabolism, and eventually the weight loss 25, 26. In this study, the deterioration of glucose metabolism and insulin sensitivity resulting from HFD in WT mice but not in CARD9^−/−^ mice was further demonstrated by glucose tolerance impairment, insulin resistance, increased blood glucose and constant insulin levels, which is consistent with previous report in CARD9^−/−^ mice 14.

HFD can increase the infiltration of liver fat cell in early and middle stage of pregnancy, especially in ApoE^−/−^ mice model 27. Our results were in accordance with previous studies that chronic HFD administration in rats would cause vacuolization and infiltration of inflammatory cells in the liver 28, 29. It was found that CARD9 resulted in pathological immune cell activation, causing inflammatory or other diseases 30. In this study, fat droplet deposition, vacuolar cells and inflammatory cells infiltration in liver tissues were observed in HFD‐treated groups, and CARD9 deficiency plays an obviously protective effect on liver damage. Moreover, the significant pathologic findings mainly the increased diameter of the adipocytes in WAT and BAT in HFD‐WT mice compared to HFD‐CARD9^−/−^ mice indicated that CARD9 knockout could ameliorate the adipocytes inflammation and fat accumulation. Along with above glucose metabolism, insulin sensitivity and energy metabolism could explain the essential function of CARD9 in diet‐induced injury.

To observe the difference of systemic inflammatory response induced by HFD in WT and CARD9^−/−^ mice, serous inflammatory cytokines IL‐6 and TNF‐α were determined. In ND‐fed group, CARD9 knockout significantly showed the reduction in IL‐6 and TNF‐α. Similarly, in HFD‐fed group, CARD9 knockout showed the reduction in IL‐6 and TNF‐α although the reduction in TNF‐α has no statistical difference between HFD‐WT mice and HFD‐CARD9^−/−^ mice, suggesting that CARD9 knockout inhibited the systemic inflammation induced by HFD. Interestingly, the consistent results also showed that the levels of TNF‐α, IL‐6 were dramatically decreased in insulin susceptive organs liver and WAT of CARD9 knockout mice.

The major objective of this study was to assess whether observed effects of CARD9 on metabolic disorders correlate with its effects on MAPKs pathway. CARD9 is selectively involved in the activation of NF‐κB and MAPKs, which are required for the production of pro‐inflammatory cytokines in innate immune responses to intracellular pathogens 4, 31, 32, 33. ERK1 and ERK2, also called p44 and p42 MAP kinases, are members of the MAPK family of proteins found in all eukaryotes. NF‐κB and MAPKs signalling pathways play pivotal roles in regulating inflammatory, immune and apoptotic responses 34. T2DM and its complications are widely believed to result from unbridled inflammatory pathways in insulin‐responsive tissues including the liver, adipose tissue, skeletal muscle and vasculature 35, 36, 37.The present study collected WAT and liver to determine the changes of CARD9/MAPKs‐related mRNA and protein expression. The results suggested that CARD9 absence is associated with the lower expression of p38, JNK and ERK1/2 in ND‐fed groups. Compared to the ND feeding, HFD feeding induced the significant elevations of p38 and JNK in WT mice but not in CARD9^−/−^ mice. Similarly, the enhancement of p38, JNK and ERK1/2 induced by HFD is lower in CARD9^−/−^ mice than that in WT mice. The results suggested that HFD induced the activation of MAPKs signalling pathway which was diminished in the absence of CARD9. Thus, it is likely that the principal effect of CARD9 knockout on ameliorating metabolic disorder and inflammation of WAT and liver is mediated by down‐regulation of the p38 MAPK signalling pathway‐mediated inflammation. This interpretation is supported by a recent finding that obesity activated p38 MAPK pathway through the up‐regulation of Bcl10/CARD9 complex 15.

The activation of NF‐κB and other transcription factors further induce the expression of various cytokines and chemokines and inflammatory responses. It is known that NF‐κB is important in the process of insulin resistance 38 and T2D 39. It has been shown that CARD9‐deficient cells are defective in zymosan‐induced NF‐κB activation 3. Meanwhile, another study also indicated that fungi‐induced NF‐κB activation through a Dectin‐2‐CARD9 signalling cascade 40. In the present study, compared to the WT mice, CARD9^−/−^ mice displayed the lower expression of NF‐κB in both HFD‐ and ND‐fed groups. Together with the lower levels of inflammatory cytokines IL‐6 and TNF‐α in serum, liver and WAT, our findings showed that the WT mice suffered a more severe injury than the CARD9^−/−^ mice after fed with HFD. The results indicated that CARD9 deficiency very likely alleviates the HFD‐induced inflammation and metabolic disorders through inactivating the MAPKs and NF‐κB signalling pathways. One recent study also strongly suggested NF‐κB and P38 MAPK molecules are activated by the up‐regulated expression of CARD9 although this study was conducted in severe acute pancreatitis 41.

The final aspect of our study demonstrated that the effect of CARD9 deficiency to enhance the expression of UCP‐1 in BAT also occurred in HFD‐fed mice when compared with the WT mice. Specifically expressed within BAT, UCP1 is largely responsible for the uncoupling of respiration from ATP synthesis, resulting in dissipation of energy as heat 42. In this study, UCP1 protein was up‐regulated in BAT of CARD9^−/−^ mice when compared to the WT mice. These results suggested that CARD9 deficiency might mediate energy metabolism and contribute to the maintenance of energy balance, which is likely associated with the enhancement of UCP‐1 in BAT. Nowadays, there is no relative study that found the association between CARD9 and UCP1. Thus, the mechanisms underlying CARD9 mediated UCP1 expression needs further exploration.

In conclusion, our results suggested that CARD9 could have a new functional role in mediating energy metabolism, insulin resistance and inflammatory response in diet‐induced inflammation and metabolic disorders, which is likely independent of the innate immunity. Thus, the CARD9 knockout‐related inhibition of NF‐κB and P38 MAPK activity is likely to be a useful approach in alleviating the inflammatory response in diet‐induced metabolic disorders. The present study suggested that CARD9 knockout may potentially ameliorate diet‐induced inflammation, insulin resistance, metabolic disorders and T2D either by inhibiting the NF‐κB and MAPKs signalling pathway or by improving insulin action and enhancing the energy metabolism.

## Conflict of interests

The authors declare that they have no competing interests.

## References

[1] Bertin J , Guo Y , Wang L , *et al* CARD9 is a novel caspase recruitment domain‐containing protein that interacts with BCL10/CLAP and activates NF‐kappa B. J Biol Chem. 2000; 275: 41082–6. 11053425 10.1074/jbc.C000726200

[2] Hara H , Ishihara C , Takeuchi A , *et al* The adaptor protein CARD9 is essential for the activation of myeloid cells through ITAM‐associated and Toll‐like receptors. Nat Immunol. 2007; 8: 619–29. 17486093 10.1038/ni1466

[3] Gross O , Gewies A , Finger K , *et al* Card9 controls a non‐TLR signalling pathway for innate anti‐fungal immunity. Nature. 2006; 442: 651–6. 16862125 10.1038/nature04926

[4] Hsu YM , Zhang Y , You Y , *et al* The adaptor protein CARD9 is required for innate immune responses to intracellular pathogens. Nat Immunol. 2007; 8: 198–205. 17187069 10.1038/ni1426

[5] LeibundGut‐Landmann S , Gross O , Robinson MJ , *et al* Syk‐ and CARD9‐dependent coupling of innate immunity to the induction of T helper cells that produce interleukin 17. Nat Immunol. 2007; 8: 630–8. 17450144 10.1038/ni1460

[6] Ruland J . CARD9 signaling in the innate immune response. Ann N Y Acad Sci. 2008; 1143: 35–44. 19076343 10.1196/annals.1443.024

[7] Jia XM , Tang B , Zhu LL , *et al* CARD9 mediates Dectin‐1‐induced ERK activation by linking Ras‐GRF1 to H‐Ras for antifungal immunity. J Exp Med. 2014; 211: 2307–21. 25267792 10.1084/jem.20132349 PMC4203953

[8] Whibley N , Jaycox JR , Reid D , *et al* Delinking CARD9 and IL‐17: CARD9 Protects against Candida tropicalis Infection through a TNF‐alpha‐Dependent, IL‐17‐Independent Mechanism. J Immunol. 2015; 195: 3781–92. 26336150 10.4049/jimmunol.1500870 PMC4592105

[9] Lee EJ , Brown BR , Vance EE , *et al* Mincle activation and the Syk/Card9 Signaling axis are central to the development of autoimmune disease of the eye. J Immunol. 2016; 196: 3148–58. 26921309 10.4049/jimmunol.1502355 PMC4799727

[10] Peterson MR , Haller SE , Ren J , *et al* CARD9 as a potential target in cardiovascular disease. Drug Des Devel Ther. 2016; 10: 3799–804. 10.2147/DDDT.S122508 PMC5125811 27920495

[11] Mandrup‐Poulsen T . IAPP boosts islet macrophage IL‐1 in type 2 diabetes. Nat Immunol. 2010; 11: 881–3. 20856216 10.1038/ni1010-881

[12] Larsen CM , Faulenbach M , Vaag A , *et al* Interleukin‐1‐receptor antagonist in type 2 diabetes mellitus. N Engl J Med. 2007; 356: 1517–26. 17429083 10.1056/NEJMoa065213

[13] Jagannathan M , McDonnell M , Liang Y , *et al* Toll‐like receptors regulate B cell cytokine production in patients with diabetes. Diabetologia. 2010; 53: 1461–71. 20383694 10.1007/s00125-010-1730-z PMC2895399

[14] Cao L , Qin X , Peterson MR , *et al* CARD9 knockout ameliorates myocardial dysfunction associated with high fat diet‐induced obesity. J Mol Cell Cardiol. 2016; 92: 185–95. 26900039 10.1016/j.yjmcc.2016.02.014 PMC4904726

[15] Wang S , Gu J , Xu Z , *et al* Zinc rescues obesity‐induced cardiac hypertrophy via stimulating metallothionein to suppress oxidative stress‐activated BCL10/CARD9/p38 MAPK pathway. J Cell Mol Med. 2017; 21: 1182–92. 28158919 10.1111/jcmm.13050 PMC5431126

[16] Xu X , Yavar Z , Verdin M , *et al* Effect of early particulate air pollution exposure on obesity in mice: role of p47phox. Arterioscler Thromb Vasc Biol. 2010; 30: 2518–27. 20864666 10.1161/ATVBAHA.110.215350 PMC3065931

[17] Huang W , Bansode RR , Bal NC , *et al* Protein kinase Cbeta deficiency attenuates obesity syndrome of ob/ob mice by promoting white adipose tissue remodeling. J Lipid Res. 2012; 53: 368–78. 22210924 10.1194/jlr.M019687 PMC3276460

[18] Katsuki A , Sumida Y , Gabazza EC , *et al* Homeostasis model assessment is a reliable indicator of insulin resistance during follow‐up of patients with type 2 diabetes. Diabetes Care. 2001; 24: 362–5. 11213893 10.2337/diacare.24.2.362

[19] Winer DA , Winer S , Chng MH , *et al* B Lymphocytes in obesity‐related adipose tissue inflammation and insulin resistance. Cell Mol Life Sci. 2014; 71: 1033–43. 24127133 10.1007/s00018-013-1486-y PMC3954849

[20] Zhu M , Nikolajczyk BS . Immune cells link obesity‐associated type 2 diabetes and periodontitis. J Dent Res. 2014; 93: 346–52. 24393706 10.1177/0022034513518943 PMC3957341

[21] Daniels SR , Jacobson MS , McCrindle BW , *et al* American heart association childhood obesity research summit report. Circulation. 2009; 119: e489–517. 19332458 10.1161/CIRCULATIONAHA.109.192216

[22] Glocker EO , Hennigs A , Nabavi M , *et al* A homozygous CARD9 mutation in a family with susceptibility to fungal infections. N Engl J Med. 2009; 361: 1727–35. 19864672 10.1056/NEJMoa0810719 PMC2793117

[23] Dorhoi A , Desel C , Yeremeev V , *et al* The adaptor molecule CARD9 is essential for tuberculosis control. J Exp Med. 2010; 207: 777–92. 20351059 10.1084/jem.20090067 PMC2856020

[24] Arimura E , Pulong WP , Marchianti ACN , *et al* Deteriorated glucose metabolism with a high‐protein, low‐carbohydrate diet in db mice, an animal model of type 2 diabetes, might be caused by insufficient insulin secretion. Eur J Nutr. 2017; 56: 237–46. 26497335 10.1007/s00394-015-1075-y

[25] Cohen D , Gonzales‐Pacheco D , Myers O . Relationships between alanine aminotransferase, serum triglycerides, body mass index and nonalcoholic fatty liver disease in an outpatient pediatric clinic population. J Pediatr Nurs. 2016; 31: 152–8. 26690717 10.1016/j.pedn.2015.10.009

[26] Docherty NG , Canney AL , le Roux CW . Weight loss interventions and progression of diabetic kidney disease. Curr Diab Rep. 2015; 15: 1–9. 10.1007/s11892-015-0625-2 26122095

[27] Sun MN , Yang Z , Ma RQ . Effect of high‐fat diet on liver and placenta fatty infiltration in early onset preeclampsia‐like mouse model. Chin Med J (Engl). 2012; 125: 3532–8. 23044319

[28] Elshazly SM . Ameliorative effect of nicorandil on high fat diet induced non‐alcoholic fatty liver disease in rats. Eur J Pharmacol. 2015; 748: 123–32. 25542756 10.1016/j.ejphar.2014.12.017

[29] Ma Z , Chu L , Liu H , *et al* Beneficial effects of paeoniflorin on non‐alcoholic fatty liver disease induced by high‐fat diet in rats. Sci Rep. 2017; 7: 44819. 28300221 10.1038/srep44819 PMC5353673

[30] Zhernakova A , Festen EM , Franke L , *et al* Genetic analysis of innate immunity in Crohn's disease and ulcerative colitis identifies two susceptibility loci harboring CARD9 and IL18RAP. Am J Hum Genet. 2008; 82: 1202–10. 18439550 10.1016/j.ajhg.2008.03.016 PMC2427314

[31] Dong C , Davis RJ , Flavell RA . MAP kinases in the immune response. Annu Rev Immunol. 2002; 20: 55–72. 11861597 10.1146/annurev.immunol.20.091301.131133

[32] Han J , Ulevitch RJ . Limiting inflammatory responses during activation of innate immunity. Nat Immunol. 2005; 6: 1198–205. 16369559 10.1038/ni1274

[33] Ashwell JD . The many paths to p38 mitogen‐activated protein kinase activation in the immune system. Nat Rev Immunol. 2006; 6: 532–40. 16799472 10.1038/nri1865

[34] Bruckner AL . Incontinentia pigmenti: a window to the role of NF‐kappaB function. Semin Cutan Med Surg. 2004; 23: 116–24. 15295921 10.1016/j.sder.2004.01.005

[35] Gregor MF , Hotamisligil GS . Inflammatory mechanisms in obesity. Annu Rev Immunol. 2011; 29: 415–45. 21219177 10.1146/annurev-immunol-031210-101322

[36] Hotamisligil GS . Endoplasmic reticulum stress and the inflammatory basis of metabolic disease. Cell. 2010; 140: 900–17. 20303879 10.1016/j.cell.2010.02.034 PMC2887297

[37] Hotamisligil GS . Inflammation and metabolic disorders. Nature. 2006; 444: 860–7. 17167474 10.1038/nature05485

[38] Barma P , Bhattacharya S , Bhattacharya A , *et al* Lipid induced overexpression of NF‐kappaB in skeletal muscle cells is linked to insulin resistance. Biochim Biophys Acta. 2009; 1792: 190–200. 19111928 10.1016/j.bbadis.2008.11.014

[39] Harte AL , Tripathi G , Piya MK , *et al* NFkappaB as a potent regulator of inflammation in human adipose tissue, influenced by depot, adiposity, T2DM status, and TNFalpha. Obesity (Silver Spring). 2013; 21: 2322–30. 23408599 10.1002/oby.20336

[40] Bi L , Gojestani S , Wu W , *et al* CARD9 mediates dectin‐2‐induced IkappaBalpha kinase ubiquitination leading to activation of NF‐kappaB in response to stimulation by the hyphal form of Candida albicans. J Biol Chem. 2010; 285: 25969–77. 20538615 10.1074/jbc.M110.131300 PMC2923990

[41] Yang ZW , Meng XX , Zhang C , *et al* CARD9 gene silencing with siRNA protects rats against severe acute pancreatitis: CARD9‐dependent NF‐kappaB and P38MAPKs pathway. J Cell Mol Med. 2017; 21: 1085–93. 27957800 10.1111/jcmm.13040 PMC5431129

[42] Ricquier D . Respiration uncoupling and metabolism in the control of energy expenditure. Proc Nutr Soc. 2005; 64: 47–52. 15877922 10.1079/pns2004408

